# Comparing DC subsets in solid tumors: what about DC3s?

**DOI:** 10.1093/immadv/ltaf021

**Published:** 2025-05-30

**Authors:** Casper J Pachocki, Marianne Boes, Alsya J Affandi

**Affiliations:** Center for Translational Immunology, University Medical Center Utrecht, Heidelberglaan 100, 3584 CX, Utrecht, The Netherlands; Department of Pediatric Infectious Diseases and Immunology, Wilhelmina Children’s Hospital, Lundlaan 6, 3584 EA, Utrecht, The Netherlands; Center for Translational Immunology, University Medical Center Utrecht, Heidelberglaan 100, 3584 CX, Utrecht, The Netherlands; Department of Pediatric Infectious Diseases and Immunology, Wilhelmina Children’s Hospital, Lundlaan 6, 3584 EA, Utrecht, The Netherlands; Department of Molecular Cell Biology and Immunology, Amsterdam UMC Location Vrije Universiteit Amsterdam, De Boelelaan 1117, 1081 HV Amsterdam, The Netherlands; Cancer Center Amsterdam, Cancer Biology and Immunology, De Boelelaan 1117, 1081 HV Amsterdam, The Netherlands; Amsterdam Institute for Immunology and Infectious Diseases, De Boelelaan 1117, 1081 HV Amsterdam, The Netherlands

**Keywords:** dendritic cells, DC3, cancer, antigen-presenting cells

## Abstract

Dendritic cells (DCs) are critical sentinels of the immune system, serving as indispensable bridges between innate and adaptive immune responses. DCs are a heterogeneous population, with subsets playing specialized roles in immune defense, tolerance, or disease development. Among these, the recently redefined DC3 subset has gained attention for its unique features and potential roles in health and disease. This review focuses on the phenotypic, functional, and developmental diversity of DC subsets—primarily DC3s—and their contributions to cancer. The tumor microenvironment (TME) in solid tumors is characterized by varying degrees of immune cell infiltration, including DCs. Within the TME, DCs play diverse roles, either promoting anti-tumor responses or facilitating immune evasion. Key subsets include conventional type 1 and type 2 DCs (cDC1s and cDC2s), as well as plasmacytoid DCs (pDCs). DC3s share certain features with cDC2s and monocytes but are distinct in their phenotype, function, and ontogeny. Functionally, DC3s can prime and activate T cells, skewing CD4^+^ T cells towards Th17 and stimulating CD8^+^ T cells with a tissue-resident memory phenotype. In cancer, their presence correlates with diverse outcomes depending on the TME: DC3 presence is linked to increased survival in patients with pancreatic ductal adenocarcinoma and oropharyngeal cancer while in non-small-cell lung cancer and melanoma it is associated with immunosuppression. The emerging understanding of DC3s highlights the complexity of DC biology and its relevance to diseases. The dynamic immunomodulatory functions of DC3s open new avenues for developing targeted therapies against cancer and immune-mediated disorders.

## Introduction to DCs and their contributions to cancer immunity

**Figure 1.**
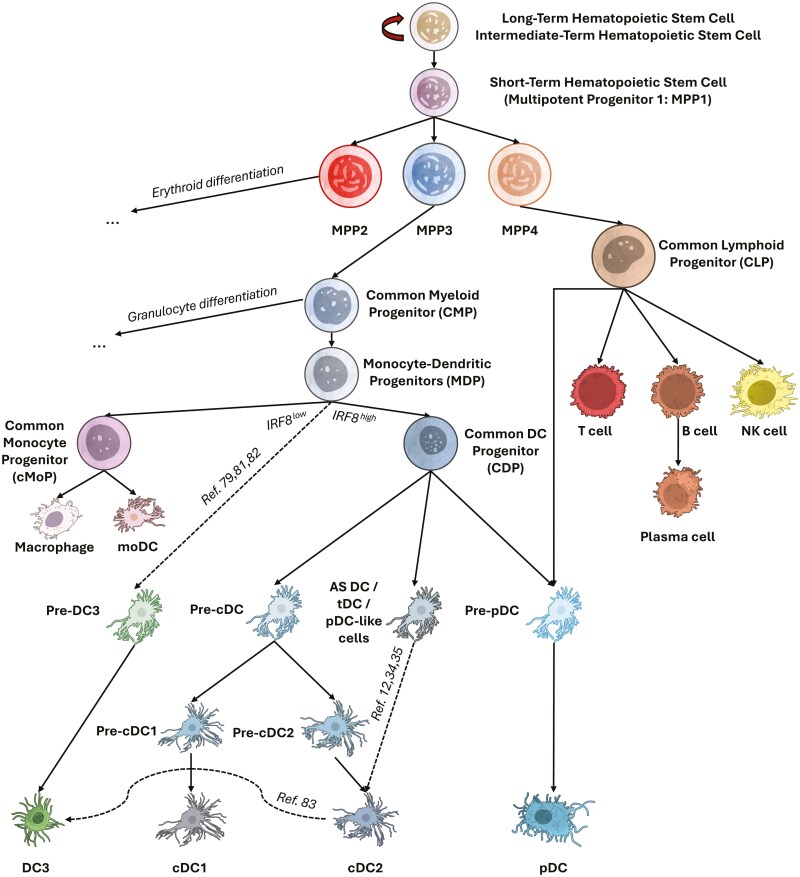
Ontogeny of human dendritic cells (DCs). Human DCs originate from hematopoietic stem cells (HSCs) in the bone marrow, which differentiate into short-term HSCs (multipotent progenitor 1 [MPP1]) and subsequently into progenitor subsets MPP2, MPP3, and MPP4, each with distinct fates. MPP2 primarily leads to erythroid differentiation. MPP4 gives rise to common lymphoid progenitors (CLPs), which develop into lymphoid cells including T cells, NK cells, B cells (and plasma cells), and plasmacytoid dendritic cells (pDCs) through pre-pDCs. MPP3 produces common myeloid progenitors (CMPs), which differentiate into granulocytes or monocyte-dendritic progenitors (MDPs). MDPs follow three pathways: under IRF8^low^ conditions, they form common monocyte progenitors (cMoPs), which generate monocytes that mature into macrophages or monocyte-derived DCs (moDCs). Alternatively, MDPs differentiate into pre-DC3 and then DC3 cells. Under IRF8^high^ conditions, MDPs produce common DC progenitors (CDPs), which give rise to pre-pDCs, AXL^+^SIGLEC-6^+^ (AS) DCs/transitional DCs, and pre-conventional DCs (pre-cDCs). Pre-cDCs further differentiate into cDC1 and cDC2 subsets through intermediates. Recent evidence suggests that cDC2 can also develop from AS DC/tDC/pDC-like lineages and cDC2 can contribute to the DC3 population. Dashed arrows indicate new/less understood pathways and are referenced by bibliographical entry. Figure was created using Microsoft Office and Adobe Illustrator. Artwork elements were obtained from NIAID NIH BIOART Source (bioart.niaid.nih.gov/bioart/).

Dendritic cells (DCs) are pivotal components of the immune system first identified by Paul Langerhans in 1868, although they were initially mischaracterized as nerve cells [1]. It was not until approximately 50 years ago that the indispensable antigen-presenting role of these cells was recognized, and their critical function in orchestrating immune responses was unearthed. In 1973, DCs were firstly isolated and characterized from mouse spleen by Ralph Steinman in Zanvil Cohn’s laboratory, revealing that this novel cell type was visually and functionally distinct from the better-known macrophages but were also highly potent stimulators of naïve T cells *in vitro* [2]. The significance of DCs in bridging the innate and adaptive immune systems has become evident, not only in promoting immune defense but also in maintaining immune tolerance to prevent autoimmunity. Furthermore, DCs are increasingly recognized for their involvement in various diseases, including infectious diseases, autoimmune disorders, and cancers. In the tumor microenvironment (TME), DCs play complex roles, influencing both anti-tumor immunity and immune evasion, making them essential to understanding and potentially treating cancer. Our understanding of DC functions, subset diversity, ontogeny, and roles in various diseases, including cancers, continues to grow significantly. An overview of DC ontogeny based on discussed literature can be found in Fig. 1.

As the complexity of DC populations continues to unfold, emerging subsets such as DC3s reveal new layers of functional diversity and developmental pathways. This raises an important question: How do different DC subsets vary in their roles across solid tumors, and what factors influence their differentiation and function in circulation and distinct TMEs? In this review, we explore the latest advancements in understanding the phenotypic and functional diversity of DC subsets, with a particular focus on DC3s, and how their interactions within the TME might shape pro- or anti-tumorigenic behavior. DC3s, in particular, have often been overlooked in the past as they share some markers and signatures with monocytes. The term ‘DC3’ has also been used to describe a DC activation state, which is not the focus of this review [3]. By assessing DC3 and other DC subsets in a variety of solid tumors, we aim to uncover how DC ontogeny and environmental cues impact their ability to modulate immune responses in cancer, providing novel insights for potential therapeutic strategies targeting these critical immune cells.

### DC function in solid tumors

DCs are unique in their capacity for antigen presentation to prime naïve T cells. In this process, DCs capture, process, and present pathogenic or tumor-derived antigens on major histocompatibility complex (MHC) molecules to T cells. DCs present antigens (such as tumor antigens) to CD8^+^ T cells via the MHCI complex, while the MHCII complex is used for priming naïve CD4^+^ T cells by extracellular peptides [4]. In addition, DCs play an important role in cytokine signaling and the inflammatory response. While DCs are known for their general roles in antigen presentation and immune modulation, they comprise highly specialized subsets that focus on or predominate certain aspects of immunity. For instance, the cDC1 subset is critical for initiating CD8^+^ cytotoxic T cell responses, while plasmacytoid DCs (pDCs) are the major interferon-producers during viral infections but are also associated with immune tolerance and the induction of regulatory T cells [5]. In recent years, the specific contributions of these DC subsets to immune regulation have been explored extensively, including their roles in the tumor context, revealing insights into their dedicated contributions.

DCs originate from precursors in the bone marrow, circulate through the bloodstream, and are actively recruited to peripheral tissues, including tumors. In the TME, each DC subset contributes differently to the balance between immune activation and immune suppression. Some DCs migrate to the (tumor-draining) lymph node to prime T cells, which has been reviewed elsewhere [6]. DCs can aid the immune response against tumors, but can also adopt tolerogenic functions, ultimately influencing tumor progression or regression. As our understanding of these specialized subsets deepens, it becomes increasingly clear that DCs are not merely passive responders but actively shape the immune landscape of the TME, with significant implications for cancer progression and treatment. Associations between DC presence in diseases and their prognosis have been described and the therapeutic targeting of DC subsets has become a topic of interest for multiple diseases, including cancers [5].

In recent years, a growing wealth of research has accumulated explaining the role of TME in tumor biology, which extends well beyond tumor-intrinsic factors that drive aberrant cell growth. Within the TME, immune cell infiltration is an important variable that predicts treatment efficacy, metastasis rate, and survival outcome [7]. Between different types of solid tumors, the level and composition of the immune cell infiltrate can vary. In tumors with high immune cell infiltration, (various types of) immune cells are abundant within the TME. Examples include melanoma, which is characterized by large numbers of CD8^+^ T cells that are indicative of a better prognosis [8]. Another example is non-small-cell lung cancer (NSCLC), where large numbers of CD8^+^ T cells, macrophages, and DCs are often found [9]. High numbers of immune cells within the TME can have varying implications. Tumors with more immune cells such as CD8^+^ T cells are more likely to respond well to treatment with immune checkpoint blockade. As these therapies rely on stimulating the immune response, immune cell-rich tumors tend to be more responsive to therapies that target immune checkpoints. While an actively responding immune system to the tumor is favorable, a chronic or exhaustive immune response could promote inflammation and facilitate tumor growth instead. Conversely, in pancreatic ductal adenocarcinoma (PDAC) and prostate cancer, the TME is notoriously immunosuppressive and low in T cells and (subsets of) DCs, contributing to their unresponsiveness to immune checkpoint inhibitors [10]. While the total immune infiltrate within the TME is a critical factor influencing cancer progression and treatment response, it has become clear that DC subsets can play pivotal roles, warranting individual appreciation for their contributions in shaping the immune landscape of these immune-variable cancers.

## Common DC subsets and activation states

### Plasmacytoid DCs (pDCs)

The pDC population is a distinct branch of DCs, originating from both the common myeloid and common lymphoid progenitor divisions. They morphologically resemble plasma cells secreting antibodies and very effectively recognize self and foreign nucleic acids through Toll-like receptors (TLRs). A hallmark of pDCs is their remarkable capacity to produce large amounts of type I interferons [11]. The development of pDCs requires high expression of IRF8, TCF4 (E2-2), and BCL11A, and likely proceeds through a pre-pDC intermediate, which in mice has been identified as a CD11c^-^Ly6D^high^ precursor population [12, 13]. Additionally, pDC differentiation involves the suppression of Id2 (Inhibitor of DNA-binding 2), which normally inhibits E2-2 [14].

pDCs can be identified as BDCA2/CD303^+^BDCA4/CD304^+^CD123^+^ cells in human, and Siglec-H^+^BST2/PDCA1^+^ cells in mice. Recently, a myeloid-derived subset called pDC-like cells was identified in mice. These cells share features with pDCs but can be distinguished by their expression of Zbtb46, and are able to differentiate into cDC2s under specific conditions [12]. Further investigation revealed that pDC-like cells are more aligned to transitional DCs (tDCs), which will be discussed below. Compared to other DC subsets, pDCs are poor APCs and do not readily prime naïve T cells, but upon activation with CD40L and IL-3, they can fully function as APCs and prime T cells [15].

In cancer, pDCs exhibit varied roles depending on their location and activation state. Circulating pDC levels are often reduced in patients with advanced-stage cancers, such as ovarian cancer, NSCLC, bladder cancer, colorectal cancer, and breast cancer [16]. However, in head and neck squamous cell carcinoma and oral squamous cell carcinoma, circulating pDC levels are comparable to those in healthy individuals. Interestingly, ovarian cancer patients experience partial recovery of circulating pDC levels after successful chemotherapy. Tumor-infiltrating pDCs generally correlate with poor prognosis in cancers like breast cancer, ovarian cancer, NSCLC, and melanoma [16, 17]. For instance, triple-negative breast cancer is characterized by an increased tumor density of pDCs, which is associated with worse outcomes. Conversely, high levels of circulating pDCs were linked to improved survival in some cancers, such as breast and pancreatic cancer, suggesting their potential as positive prognostic markers in certain contexts. These observations highlight how the location and activation state of pDCs shape their impact on cancer progression.

### Monocyte-derived DCs (moDCs)

Monocyte-derived dendritic cells (moDCs), also known as inflammatory DCs, are a distinct subset of DCs that arise from monocyte progenitors rather than from the committed DC progenitors in the bone marrow. Unlike other DC subsets, moDCs develop in response to inflammatory signals rather than through steady-state DC differentiation. Under conditions of infection, tissue injury, or other inflammatory cues, circulating monocytes (CCR2^high^ in mice, CD14^+^ in humans) infiltrate affected tissues and differentiate into moDCs, a process primarily driven by IFN-γ and other pro-inflammatory cytokines, such as GM-CSF, IL-4, and TNF-α [18, 19]. This monocytic and inflammation-dependent lineage has led to debate over whether moDCs are “*bona fide*” DCs in an ontogenetic sense; however, their functional and phenotypic similarities to other DC subsets have led to their classification within the broader DC family. Phenotypically, mouse moDCs are CD11c^high^, MHC class II^high^, and CD11b^+^, and human moDCs express CD14, CD11c, CD64, and CD88 [20, 21]. Functionally, they exhibit robust antigen-presenting capabilities, are potent stimulators of CD4^+^ T cell proliferation and polarization, and engage in cross-presentation of exogenous antigens on MHC class I complexes to CD8^+^ T cells [22].

As moDCs are rapidly recruited and differentiated under inflammatory conditions, they are predominantly found in tissues experiencing acute immune responses, including cancerous tissues. MoDCs were the first DCs to be used for tumor therapy. As they are easily obtained from monocytes upon culture with GM-CSF and IL-4, moDCs were loaded with tumor or synthetic antigens *in vitro*, before administration in patients. Only with the maturation of these loaded moDCs with inflammatory cytokines (e.g. TNF-α, IL-6) clinical outcomes and survival rates increased significantly [23]. It was expected that after administration these moDCs directly prime T cells but only a minority survive and migrate to lymph node to activate T cells [24–26]. A murine study showed that host DCs take up tumor antigens from administered moDCs, which then elicit their own immune response [27]. Therefore, the treatment setup shifted from using just moDCs to multiple types of DCs as obtained by leukapheresis from patients with castration-resistant prostate cancer. Currently, this is the only FDA-approved DC therapy, sold under the name PROVENGE [28]. For those reasons, research into DC vaccines has shifted away from moDCs to the broader DC family [25, 26, 29]. Still, moDCs might hold promise: in mouse melanoma models, the absence of moDCs greatly limits immunotherapy [30], while in pancreatic cancer moDCs are known to promote metastasis [31].

### Pre-cDCs

In the bone marrow, another DC-committed population arises from the common DC progenitor (CDP) population called pre-cDCs, which terminally differentiate into cDC1s and cDC2s in peripheral organs [32]. Pre-cDCs are therefore crucial for sustaining the population of cDCs under both homeostatic and inflammatory conditions across peripheral tissues and lymphoid organs.

Human pre-cDCs are generally characterized by CD135 (FLT3), CD117 (c-Kit), and negative for CD115 (M-CSF receptor) [32]. Pre-cDCs are a heterogeneous population that can be found in bone marrow and blood, with pre-commitment to become CD141^+^ cDC1 or CD1c^+^ cDC2, based on their CD172 expression [33]. More recently, three subsets of pre-cDCs were identified: CD123^+^CADM1^−^CD1c^−^ uncommitted pre-DC, as well as CADM1^+^CD1c^−^ pre-cDC1 and CADM1^−^CD1c^+^ pre-cDC2 (13). This seemed to be just the first glimpse into the complexity of this previously called pre-cDC population. The subsequent identification of other DC subtypes, ontogenetically related to the earlier presumed pre-cDC population, revealed that pre-cDCs are not merely precursors to cDC1/2s, but have distinct roles and functionalities.

### AS DCs/tDCs

At the time pre-DCs were described, independently, a DC subset expressing AXL, SIGLEC1, and SIGLEC6 (AS DCs), was identified as a distinct DC population that arises from the CDP progenitor. They share markers and gene expression signatures with pDCs and can differentiate into cDC2s [34]. As AS DCs (also called AXL^+^ DCs) are also able to prime T cells but do not proliferate, it is hypothesized that instead of a precursor, they represent a distinct functional DC subset that can transition to cDC2. There are clear similarities between pre-DC and AS DC, as they express AXL and SIGLEC6 in both studies.

In the search for the pre-DC/AS DC murine homolog, a pDC-like population was found that comprises pDC and cDC2 characteristics but does not express Axl [35]. To align the murine and human populations, they are now often referred to as transitional DCs (tDCs), a distinct population from cDCs, sharing properties with pDCs, and potentially contributing to the broader cDC2 population [36].

Although research on AS DCs in disease remains limited, recent studies have shown that increased numbers of AS DCs were linked to CNS autoimmunity [37], and AS DCs are also recruited during cutaneous inflammation [38, 39]. They can be found in the circulation of patients with various types of cancer, including pancreatic cancer and melanoma [40, 41]. Currently, AS DCs or tDCs remain an understudied population and their function and exact role in disease pathogenesis remains unclear: whether they represent a terminally differentiated state, a precursor to cDC2s, or are a distinct subset with inflammatory or regulatory functions [36].

### Conventional DC1s (cDC1s)

The cDC1 population is one of the most clearly understood DC branches and is crucial for cross-presentation, which initiates CD8^+^ T cell responses. The generation of cDC1 cells (rather than cDC2s) from pre-cDCs depends on specific transcription factors, primarily IRF8 and BATF3 in mice [42], which work together to drive gene expression towards the cDC1 lineage. The cDC1 population is known for very effective antigen presentation of necrotic cells, induction of viral responses, and antitumor immunity [43].

Also referred to as CD141/BDCA3^+^ DCs in humans, cDC1s can be further divided into two main populations in mice: CD8α^+^ cDC1s which reside in lymphoid tissue, and CD103^+^ cDC1s which are migratory cells. Both fractions participate in cross-presentation on MHC class I to CD8^+^ T cells but have some distinct properties. For example, CD103^+^ DC1s bring tumor antigen to the draining lymph node, have a greater dependence on GM-CSF, and are more potent inducers of a Th17 response [44]. While CD141 is a key marker for cDC1s in human, it is not unique to this subset [45]. Additional markers that help to define cDC1s include CLEC9A (C type lectin receptor 9A; also known as DNGR-1) [46], CADM1 [47], BTLA (CD272) [48], and (after terminal differentiation) XCR1 [49]. Among these, CLEC9A is particularly important as it mediates the presentation of antigens from dying cells [50–52]. Additionally, the unique expression of the XCR1 receptor marked more mature cDC1s with potent antiviral capabilities [49].

Especially in cancer immunity, increased presence and activity of cDC1s was associated with increased survival in multiple tumors [53]. In tumor tissues, CD103^+^ cDC1s express CXCL9 and CXCL10 that attract activated T and NK cells through CXCR3 [54]. Similar to other DCs they upregulate CCR7 after antigen internalization, playing a critical role in inducing migration towards lymphoid organs for T cell priming [55, 56]. Compared to healthy controls, advanced melanoma patients had fewer circulating CD141^+^ cDC1s, which also showed impaired responses upon TLR stimulation *ex vivo*, particularly in patients who did not respond to checkpoint blockade treatment [57]. Although there was no direct association with response to anti-PD-1 treatment, injections of CD141^+^ cDC1s into the tumor in a humanized mouse model attenuated tumor growth in combination with anti-PD-1 treatment. Similarly, intratumoral expansion and activation of CD103^+^ cDC1s can improve the effectiveness of anti-PD-1 treatment [58]. In PDAC, pancreatitis often precedes tumor development. During pancreas inflammation, cDC1s are recruited and promote activation of CD4^+^ regulatory T cells over cytotoxic CD8^+^ T cells, likely to prevent tissue damage. In a mouse model of advanced PDAC coupled with pancreatitis, these inflammation-recruited cDC1s sensitize PDAC to immune checkpoint blockade [59]. Although cDC1s are rare in human PDAC tissue, their presence is correlated with longer survival. Furthermore, in the KPC mouse model, Flt3 ligand (Flt3L)-mobilized cDC1s and cDC2s lead to reduced desmoplasia, increased T cell activation, and response to radiotherapy [60]. While cDC1s are essential to antitumor immunity due to their unique ability to cross-present antigens to cytotoxic CD8^+^ T cells [61], their broader role during tumor development seems to be more complex, and a better understanding is needed to determine the optimal therapeutic window.

### Conventional DC2s (cDC2s)

In lymphoid organs, cDC2s (also known as CD1c^+^ DCs) are typically the most prevalent subset of conventional dendritic cells (cDCs). Unlike the relatively homogeneous cDC1 population, cDC2s are more diverse, dependent on IRF4, and are generally characterized by the expression of CD11b in mice [62]. The transcription factor Zeb2 is crucial for cDC2 development by repressing Id2, thereby directing commitment from the CDP to the cDC2 lineage [63, 64]. In contrast to cDC1s, which primarily drive cytotoxic T cell responses, cDC2s are more inclined to prime naïve CD4+ T cells towards a Th2 or Th17 phenotype [65].

Conventionally, cDC2s are separated from cDC1s based on the expression of CD1c (BDCA1). Over the last years, researchers worked on aligning separate populations from the heterogeneous cDC2s. Two subsets of cDC2s can now be discriminated based on CD5 expression [66]. The CD5^high^ population preferably induced naïve T cell proliferation and T cells producing IL-4 (Th2 response), while the CD5^low^ population resulted in higher levels of T cells producing IFN-g. Another study found another cDC2 subdivision in mice based on Tbet expression, that created a cDC2A (Tbet^+^, involved in tissue repair and less induced to polarize T cells) and a cDC2B (Tbet^-^, more pro-inflammatory) subtype. In humans, these are equivalent to CD1c^low^CLEC10A^–^CLEC4A^high^ and CD1c^+^CLEC10A^+^CLEC4A^low^ cDC2s (Tbet^+^ and Tbet^-^, respectively) [67].

While the former subtypes were found based on ontology, an inflammatory cDC2 (inf-cDC2) phenotype was discovered in mice undergoing a respiratory infection. Interestingly, the authors describe that this inf-cDC2 cell state, expressing a *bona fide* cDC marker CD26, induced CD4^+^ T cells and cross-presented to CD8^+^ T cells. The typical CD11c^+^MHC-II^+^CD11b^+^ moDCs, but low expression of CD26, have poor migratory and APC potentials [68]. A widely accepted idea is that the diversity observed within cDC2 populations stems from their transcriptional adaptation to specific tissue environments, which determine their roles in promoting either immune tolerance or active immunity [69]. Based on the discovery of the inf-cDC2 state, understanding whether the various cDC2 subsets represent distinct lineages or alternative activation states has become critical, just as uncovering the specific T-cell differentiation programs they initiate.

In PDAC, many factors influencing T cells and DCs were described that contribute to the observed low responsiveness to immunotherapy. Specifically, a recent study showed that low levels of cDC2s correlated with worse patient outcomes [70]. Looking at the role of cDC2s in melanoma, it was found that a high degree of cDC2 (BDCA1^+^) cells correlated with the number and activation state of CD4^+^ T cells, in turn linked to increased responsiveness to anti-PD-L1 therapy [71]. Interestingly, the role of cDC2 cells depended on the suppressive capacity of Tregs, and only then cDC2 presence correlated with therapy responsiveness. Altogether, these two examples indicate that the role of cDC2s in solid cancers is multi-factorial.

### Lineage-unrestricted mregDCs

Traditionally, the classification of dendritic cell (DC) subtypes occurs based on their lineage, which has been instrumental in understanding their phenotypes and functions. However, it is also possible that certain DC states arise independently of lineage, driven instead by shared environmental factors. This idea is supported by evidence showing that DCs exposed to similar external stimuli can adopt comparable functional programs, regardless of their origin. For instance, mature regulatory DCs (mregDCs) can be identified as a conserved state across various DC subtypes, characterized by specific regulatory functions likely shaped by environmental cues rather than lineage commitment. A cluster of DCs resembling classical cDC1s and cDC2s was identified, expressing immunoregulatory and maturation genes in the same program [72]. Interestingly, this mregDC state reduces cDC1’s ability to cross-present, thereby limiting anti-tumor immunity. Already, combining immunotherapy with Wnt pathway inhibition has been shown to upregulate mregDCs in a mouse model for glioblastoma, helping to overcome resistance to immune checkpoint blockade [73]. With accumulating evidence for its role in anti-tumor immunity [74], therapeutic targeting of mregDCs might therefore prove valuable for the treatment of solid tumors.

Another study explored the role of stromal cells in the transition of self-renewing TCF-1^+^ cytotoxic T cells towards the more proliferative TCF-1^-^ effector-like state in a mouse melanoma model. Over time, these effector cells may lose proliferative capacity and functionality and adopt a hypofunctional state, limiting anti-tumor immunity. The authors found that an intratumoral “DC3 state” provides critical survival signals to rescue the effector-like T cells from activation-induced cell death, thereby maintaining their anti-tumor response [75]. This “DC3 state” closely aligns with the previously discussed mregDC definition [72], are also called LAMP3^+^ DCs [76, 77] or CCL22^+^ cDC1s [78], and can easily be confused with the more widely accepted definition of DC3 cells as a distinct developmental subset of DCs which is discussed below. In this review, we specifically focus on the DC3 cell type and not the DC3 state.

### A newly redefined DC population: DC3s

With the arrival of advanced technologies such as single-cell RNA sequencing (scRNA-seq), the full heterogeneity of DC populations is being explored in unprecedented detail. Previously, DC subsets were primarily characterized through methods that focused on surface markers, functional assays, and microscopy. While these methods laid the foundation for understanding DC biology and identified the larger subsets, they seem to have missed the finer distinctions within each subset. Roughly 15 years ago, bulk RNA sequencing revolutionized the field by allowing to capture large-scale gene expression in a population, uncovering broad transcriptional differences between known defined DC populations. As technology progressed, scRNA-seq now equips researchers with the ability to resolve transcriptional differences between single cells, dramatically increasing the resolution of the approach. In turn, this enables the identification of very rare and previously unknown DC subsets in unbiased manner, impossible by traditional methods. However, the lack of standardization in scRNA-seq methodologies has also led to increased confusion in the field regarding DC subset classification and functional characterization. Differences in data processing, clustering algorithms, and marker selection can result in varying conclusions about the identity and roles of specific DC populations. Nevertheless, identifying subsets within larger DC populations, such as cDC2, could provide insight into their diverse functions in diseases. The roles previously attributed to the cDC2 population might instead root from the presence of multiple smaller or cDC2-related subsets, each with unique functions and specific contributions to anti-tumor or tumor-promoting immunity.

In one of the first instances that applied scRNA-seq for the identification of DC and monocyte subsets, six human DC subtypes and four monocyte subsets were classified in blood, among which AS DCs, as well as a DC subset that they named DC3 (34). As this DC3 population resembles cDC2s due to its CD1c expression, additional markers to distinguish for cDC2s (CD32B) and the novel DC3 population (CD163, CD36) were identified. Both subtypes potently activated naïve T cells, but cDC2s expressed immunomodulatory cytokines CCL19, IL-10, IL-12B, and IL-18 at higher levels. On the other hand, this DC3 population had robust expression of (generally monocyte-related) genes linked to inflammation, such as CD14, S100A9, and S100A8. As CD14 and other related markers are commonly expressed by monocytes, one could then ask: are DC3s monocytes or DCs?

In a second example, high dimensional protein expression and scRNA-seq techniques were combined with machine learning and pseudo-time analysis to explore DC heterogeneity. Using their own high-dimensional single-cell protein expression pipeline, they isolated a cDC2 population, which with subsequent analysis revealed a great presence of CD14, CD5, and CD163 [79]. They then identified a DC3 population but used CD5 expression, rather than CD163^+^ and CD14^+^ to distinguish cDC2s from DC3s [79]. The authors suggested that from a CD5^+^ population with pre-DC properties, they differentiated towards three DC3 subsets in stages: CD5^−^CD163^−^, CD5^−^CD163^+^CD14^−^, and CD5^−^CD163^+^CD14^+^ populations. The latter consisted of the largest cells among cDC2 and DC3 compartments and displayed DC-like membrane characteristics, specifically a rougher, more granular texture. Then, to confirm that DC3s are ontogenically related to cDC2s and not monocytes, the authors treated patients with FLT3L. FLT3 plays a crucial role in DC specification by promoting the differentiation and expansion of DCs from both lymphoid and myeloid progenitors in response to FLT3L [80]. While all cDC2-related subsets are dependent on FLT3L, the common monocyte progenitor (cMoP) lineage is not. With FLT3L treatment, the numbers of all cDC2-related subsets (including DC3s) were increased, but the CD1c^-^CD14^high^ classical monocyte population was reduced [79]. This directly indicates that while this novel DC3 population expresses monocyte-related genes, it does not directly stem from a monocyte precursor. However, further validation through experimental data was needed to confirm the developmental model and the predicted cell trajectories.

Other groups have since refined the lineage of DC3 development by showing that instead of developing from a cDC2 population, DC3s stem from a small portion of granulocyte-monocyte and DC progenitor cell population (CD34^+^CD38^+^CD45RA^+^) that is IRF8^low^, CLEC12A^+^, and has minimal expression of CD123 [81, 82]. How well these IRF8^low^ and CLEC12A^+^ DC3 progenitors are aligned is yet to be determined. Additionally, GM-CSF was found to be necessary for DC3 differentiation from CD34^+^ progenitors on a stromal cells-supported system, while FLT3L was not required [81]. Using GM-CSF, they could not demonstrate a transdifferentiation from cDC2s into DC3s, something that was suggested earlier [79]. While the combined evidence clearly delineates a separate developmental lineage for DC3s, another study showed that CD1c^+^CD14^-^ cDC2s could be converted towards CD1c^+^CD14^+^ DC3s through IL-6 and M-CSF [83]. While bona fide DC3s therefore have a separate developmental lineage, the alternative possibility of cDC2s transdifferentiating into DC3s cannot be ruled out.

**Figure 2.**
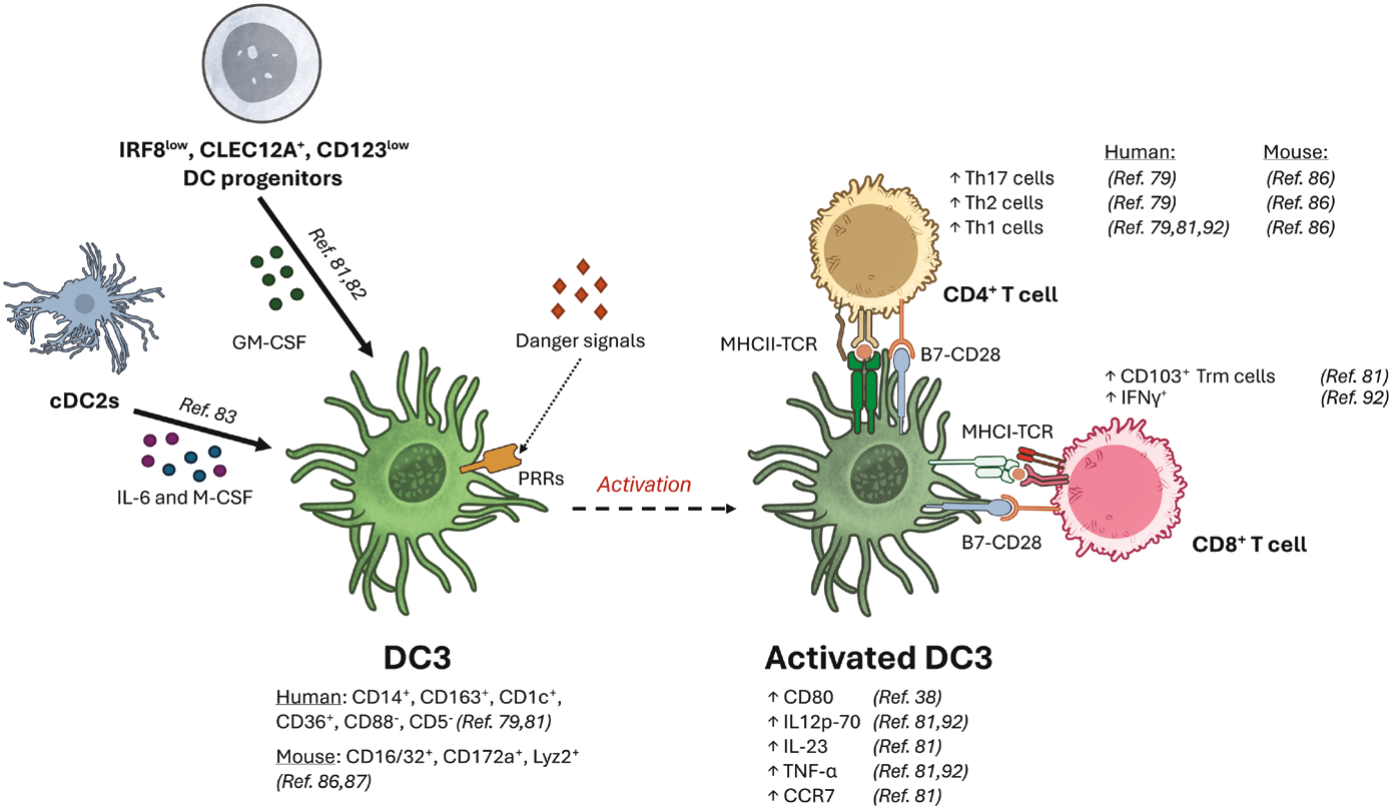
*DC3 development and function.* Human DC3s develop from IRF8^low^, CLEC12A^+^, CD123^low^ DC progenitors in response to GM-CSF. Alternatively, they can transdifferentiate from cDC2 under the influence of IL-6 and M-CSF. Upon stimulation of pattern-recognition receptors (PRRs) by danger signals, DC3s transition into an activated state marked by increased expression of maturation marker CD80 (B7-1), CCR7, as well as cytokines IL-12p70, IL-23, TNF-α, among others. Activated DC3s are known to activate T cell responses with skewing towards Th17 and promoting CD8^+^ CD103⁺ tissue-resident memory T cells (Trm). Figure was created using Microsoft Office and Adobe Illustrator. Artwork elements were obtained from NIAID NIH BIOART Source (bioart.niaid.nih.gov/bioart/).

The DC3 population is currently characterized by the expression of CD1c, CD14, and the absence of CD88. While all monocytes are CD88^+^, cDC2s are CD88^-^CD1c^+^CD14^-^ (with or without CD5) [84] and DC3s are CD88^-^CD1c^+^CD14^+^CD163^+^. DC3s share phenotypic markers with cDC2s and monocytes, and this population also exhibits functional similarities to both cell types. Various studies have assessed the functional role of DC3s in T cell priming in an allogeneic setting. Naïve T cell polarization was compared after co-culture with the cDC2 or DC3 subset, revealing that while Th1 polarization was similar across subsets, Th2 and especially Th17 CD4^+^ T cells priming was increased by DC3s, especially the CD5^−^CD163^+^CD14^+^ subset [79]. However, in another study, only IFN-γ and TNF-α- producing CD4⁺ T cells were found after co-culture with DC3, perhaps due to the exclusion of PMA ionomycin restimulation in the latter [81]. Additionally, the DC3 subset was found to be particularly effective at driving the differentiation of naïve CD8⁺ T cells into CD8⁺CD103⁺ tissue-resident memory T cells through TGF-β, but only after treatment with a cocktail of TLR-agonists [81]. Also, activated DC3s were shown to upregulate CCR7, a feature of cDC2s, and secrete high levels of cytokines typical of cDC2s, such as IL-12p70 and IL-23, which are important for T cell priming and inflammation. Additionally, DC3s produced TNF-α, more commonly associated with monocytes, and upon IFNβ-exposure they expressed a high amount of co-stimulatory molecule GITRL [85]. It therefore seems that not only phenotypically but also functionally, DC3s overlap with cDC2s and monocytes. An overview of human DC3 development and function can be found in Fig. 2.

### Mouse DC3s

The recent identification of DC3 cells among human DC subsets raised the question of whether analogous populations exist in mice. Using multimodal profiling, a lineage of murine CD16/32^+^CD172a^+^Lyz2^+^ DC3s, distinct from cDC2s, was identified. Similar to human DC3 development (Fig. 1), in mice, DC3s arise from a Ly6C^+^ MDP progenitor through a Lyz2^+^Ly6C^+^CD11c^-^ pro-DC3, whereas cDC2s develop from CDP progenitors through CD7^+^Ly6C^+^CD11c^+^ pre-DC2s [86]. Functionally, DC3s showed comparable CD4^+^ T cells activating capacity to cDC2s, with skewing towards Th17 phenotype, underscoring previous findings in humans [79]. In addition, this FcγRIIB/III(CD16/32)^+^ putative DC3 population was found to have a widespread distribution across major murine tissues and could express CD301b and Clec12A [87]. As the murine DC3 population remains poorly characterized, further lineage tracing, transcriptomic profiling, and functional studies are required to define its features and functional properties.

### DC3s in disease

The role of DC3s in disease remains challenging to elucidate, largely due to the relatively recent establishment of consistent markers for identifying this population. Before the classification of DC3s, CD1c⁺CD14⁺ DCs were reported in various tumor contexts, also often referred as inflammatory DCs [88]. For instance, in breast cancer, the transcriptional profiles of infiltrating CD1c⁺CD14⁺ DCs and other DC populations were extensively studied, revealing their subset-specific adaptation to the TMEs of different breast cancer types [89]. However, it remains unclear whether these previously identified populations are true DC3s or distinct subsets of cDC2s or monocytes. Although CD14 expression is lower in DC3, a proper monocyte marker such as CD88 should be included.

A consistent definition of DC3s remains a significant challenge, as their discovery relies on high-resolution techniques like scRNA-seq, which are low throughput and prone to variability. Even with the addition of other markers such as CD163, difficulties persist, as these markers can be limited by factors like susceptibility to enzymatic degradation [81]. To reliably identify and characterize DC3s in future studies, it will be essential to integrate multiple approaches. These may include combining transcriptomic profiling with protein-level analyses, spatial mapping within tissues, and functional (T cell) assays that assess their unique contributions in disease contexts. Such comprehensive strategies will be critical for distinguishing DC3s from other closely related populations and for understanding their roles in the dynamic environments of health and disease.

Since the establishment of DC3s as a *bona fide* DC subtype, its role in disease pathogenesis was first examined in autoimmunity. One study revealed that CD163⁺CD14⁺ DC3s were significantly increased within the CD5⁻ DC subset exclusively in systemic lupus erythematosus (SLE) patients which correlated with disease severity [79]. They displayed elevated secretion of pro-inflammatory cytokines especially in active SLE patients. These findings emphasize the need to investigate the DC3 population in different pathological conditions. Indeed, more recent studies suggest that DC3 is also involved in other inflammatory conditions, which are beyond the scope of this review [90, 91].

Gradually, the role of DC3s in solid tumors is being elucidated. In HPV16-positive oropharyngeal cancer, CD163^+^ DC3s support antitumor immunity by driving CD4^+^ and CD8^+^ type I responses and cytokine production, associated with tumor-specific T cell infiltration and improved patient survival [92]. In recent immune profiling studies of PDAC patients, we observed an increased frequency of DC3s in the blood [40], and their presence within the tumor was associated with higher survival [93]. Similarly, DC3s were increased in Luminal A-type breast cancer, indicating potential sensitivity to immunotherapy [94]. Although DC3s infiltration in breast cancer did not correlate with disease progression or other DC subsets, DC3s positively correlated with the frequency of CD8⁺CD103⁺ tissue-resident memory T cells [81]. More studies on the role of DC3s in solid tumors with a larger cohort of patients will help to clarify DC3 functions in cancer.

Many cancers exploit mechanisms to evade immune detection and suppress anti-tumor immune responses, leading to insufficient immune activation against tumor cells. In a study assessing melanoma and NSCLC, both tumors with high immune infiltration, researchers found that non-mature CD1c^+^ CD14^-^ cDC2s could be directed towards DC3s (CD1c^+^ CD14^+^ cDC2s) through tumor-derived IL-6 and M-CSF (CSF1) [83]. This DC3 conversion (increased expression of CD14 by cDC2) was also observed in a co-culture with colorectal cancer organoids, which was partially mediated by IL-6 and PGE-2 [95]. Additionally, they showed that CD14^+^ cDC2s cells from melanoma patients had decreased T cell proliferation and activating capacity compared to canonical cDC2s [96], and blood CD14^+^ cDC2s (*de facto* DC3s) numbers relate to lower survival rates. Interestingly, healthy donor CD14^+^ cDC2 cells could be reverted to CD14^-^ cDC2s under the influence of TNF-a and IL-1b, but not after exposure to TME-derived factors. From these data, the authors conclude that DC3s can be transdifferentiated from cDC2 and contribute to the low immunostimulatory environment in melanoma and NSCLC. Of note, monocytes were only excluded based on CD14^high^CD1c^-^ expression in these studies, and whether they truly represent a bona fide DC3 population remains to be determined. As data across studies and tumors remain variable, further research into the role of DC3s in solid tumors is warranted. An overview of the discussed literature on DC3 cells can be found in Table 1.

**Table 1.** Table of summary of discussed DC3 findings.

Conclusion	Clinical findings	Reference
DC3s represent a unique population that shares expression patterns with both cDC2s and monocytes.	n.d.	[34]
CD88^-^CD1c^+^CD5^-^ DC3s can be divided into three subsets: CD5⁻CD163⁻ cells, CD163⁺CD14⁻ cells, and CD1c^low^CD163⁺CD14⁺ cells.	Increased CD14⁺ DC3 levels are associated with heightened disease activity in systemic lupus erythematosus.	[79]
CD88^-^CD1c^+^CD14^+^ DC3s originate from a minor fraction of granulocyte-monocyte and DC progenitor cells characterized by IRF8^low^, CLEC12A⁺ expression, and low levels of CD123.	Increased DC3 numbers are linked to the expansion of tissue-resident T cells and improved prognosis in breast cancer.	[81, 82]
IL-6 and M-CSF can reprogram CD1c^+^CD14^-^ cDC2s to differentiate into CD1c^+^CD14^+^ DC3s.	Elevated CD14⁺ DC3 counts correlate with decreased survival in non-small cell lung cancer and reduced survival rates in melanoma.	[83]
CD1c^+^CD14^-^CD163^+^ DC3s induce T cell proliferation and stimulate Th1 polarization.	High numbers of DC3s correlate with improved survival in HPV16-induced oropharyngeal cancer.	[92]
CD88^-^CD1c^+^CD14^+^ DC3s are found in pancreatic ductal adenocarcinoma tumors.	Higher DC3 counts are associated with improved survival in pancreatic ductal adenocarcinoma.	[93]
CD1c^+^CD14^+^ DC3 frequency is positively associated with the presence of tissue-resident memory CD8⁺ T cells in Luminal A-type breast cancer.	Elevated DC3 numbers are linked to better survival outcomes in Luminal A-type breast cancer.	[94]

These initial studies provide a foundation for future research to clarify the precise role of DC3s in shaping the TME. As research continues to uncover the mechanisms by which DC3s influence immune responses, their dual potential to either support anti-tumor immunity or contribute to immune suppression highlights their promise as both biomarkers and therapeutic targets in specific solid tumors, although further validation is needed. The pro- and anti-tumorigenic behavior of DC3s highlights the unique ways in which different cancers exploit the host’s immune system, adding yet another layer of complexity to the challenge of treating solid tumors. This emerging knowledge marks the beginning of a deeper understanding that could pave the way for novel therapeutic strategies targeting DC3s to enhance anti-tumor immunity.

### Concluding remarks and future directions

The identification of distinct DC subtypes and states in humans and mice is expected to expand significantly with the continued refinement of advanced high-resolution techniques including scRNA-seq. These technological advances may render previously characterized populations outdated, leading to their reclassification into smaller, more homogeneous subsets. However, the lack of standardization in scRNA-seq methodologies, such as different clustering algorithms and marker selection, can result in varying conclusions about the identity and roles of specific DC populations. While such refinements enhance our comprehension of fundamental aspects of DC ontogeny, distribution, and function, they also highlight the pressing need for a standardized and consistent classification framework for DC populations. Future studies should integrate multi-omics approaches, combining transcriptomics, proteomics, and epigenomics along with spatial data and computational modeling to establish a robust and accurate foundation for DC subset stratification across different studies. This foundational framework will ensure consistency and comparability between research, allowing for optimal use of data.

Next, it is equally important to consider how distinct environments influence DC roles and functions, particularly in diseases such as cancer and chronic inflammation. As demonstrated in this review, the diverse microenvironments created by different tumor types profoundly shape the roles and behavior of DC subsets, as best exemplified by mregDCs. However, research in this area is complicated by two primary challenges: the lack of consensus on DC classification as discussed above and the limited availability of patient material for study. Moreover, the focus on T cells in cancer immunology often overshadows the equally crucial roles of DCs. Despite these challenges, evidence of distinct DC roles in cancer has already emerged, emphasizing the need for future clinical studies to functionally assess the interplay between DCs, T cells, and tumor progression. For this, the emergence of spatial transcriptomics technologies should help us answer these questions. Additionally, rigorous lineage tracing studies could help clarify differentiation and ensure consistency in identifying DC3 populations across studies.

Building on clinical evidence thus far, functional studies of DCs within TMEs are critical to advancing our understanding of the DC-tumor crosstalk. This research should aim to dissect the signaling pathways and environmental cues that dictate DC behavior in cancer and other disease contexts. By applying approaches to more clearly defined DC subsets, researchers will be better equipped to investigate these interactions and identify novel therapeutic avenues for solid tumors and beyond. *Bona fide* DC3s differentiated from CD34^+^ progenitors or induced pluripotent stem cells could further advance the study of these interactions, providing valuable models for identifying and validating new therapeutic targets, as well as developing cell-based therapies for cancer and other diseases [97, 98].

In conclusion, while much remains to be discovered, knowledge of DC biology is expanding rapidly. DC3s highlight the remarkable pace of this progress: within just seven years, research has identified two distinct developmental lineages and three functionally unique DC3 subsets. Clinical studies so far reveal the variability in DC3 roles across different tumor types, emphasizing both their complexity and therapeutic potential. These advancements show the potential for future research to uncover additional DC subtypes, developmental pathways, and context-specific functions. With coordinated efforts bridging fundamental, clinical, and translational research, we can continue to deepen our understanding of DC biology, ultimately paving the way for transformative treatments for a range of diseases.

## Permission to reproduce

Figures 1 and 2 were created using Microsoft Office and Adobe Illustrator. Artwork elements were obtained from NIAID NIH BIOART Source (bioart.niaid.nih.gov/bioart/).
